# Association of the *CYP19A1* rs700518 Polymorphism with Selected Markers of Bone Metabolism in Women with Hyperandrogenism

**DOI:** 10.3390/jcm11123537

**Published:** 2022-06-20

**Authors:** Izabela Uzar, Anna Bogacz, Elżbieta Sowińska-Przepiera, Krzysztof Piątek, Filip Przerwa, Marlena Wolek, Bogusław Czerny

**Affiliations:** 1 Department of Pharmacology and Pharmacoeconomics, Pomeranian Medical University, 71-230 Szczecin, Poland; uzari@wp.pl (I.U.); fmprzerwa@gmail.com (F.P.); boguslaw.czerny@iwnirz.pl (B.C.); 2 Department of Stem Cells and Regenerative Medicine, Institute of Natural Fibers and Medicinal Plants, 62-064 Plewiska, Poland; marlena.wolek@iwnirz.pl; 3 Department of Endocrinology, Metabolic Diseases and Internal Diseases, Pomeranian Medical University, 71-252 Szczecin, Poland; elzbieta.sowinska@pum.edu.pl; 4 Department of Gynecology and Obstetrics, Collegium Medicum, University of Zielona Góra, 65-001 Zielona Góra, Poland; krzysztofpiatek.jr@gmail.com

**Keywords:** hyperandrogenism, gene polymorphism, CYP19A1, bone metabolism

## Abstract

Hyperandrogenism is the most common endocrine disorder in women, characterized by an imbalance of normal estrogen and androgen levels in the blood. Androgens play an important role in the female body because they influence bone mineral density (BMD), body mass composition, muscle mass, mental state, and the regulation of sexual function. The reduced activity of aromatase, due to mutations in the *CYP19A1* gene, reduces the estrogen pool in favor of androgens. Clinically, aromatase deficiency causes hyperandrogenism in women. Therefore, the aim of the study was to assess the effect of the *CYP19A1* gene polymorphism on selected markers of bone metabolism and hormonal parameters in women with hyperandrogenism. The study group was comprised of 80 young women with hyperandrogenism who underwent measurements of bone mineral density (BMD), and determination of hormonal and metabolic parameters. Enzyme immunoassays were used to measure leptin, total sRANKL (free and bound RANKL), osteoprotegerin, and total 25-OH Vitamin D. An analysis of the *CYP19A1* gene polymorphisms was performed using the real-time PCR method. The GG genotype of the *CYP19A1* rs700518 polymorphism turned out to be associated with: FEI (Free Estradiol Index), SHGB concentration, estradiol concentration, and insulin concentration determined in the glucose tolerance test 60’ compared to AG and AA genotypes. Patients with the AG genotype had a higher ratio of android to gynoid fat and a greater content of visceral adipose tissue. Higher visceral tissue content may reduce BMD. In conclusion, the study showed that the *CYP19A1* rs700518 polymorphism may be associated with the distribution of adipose tissue in young women with hyperandrogenism. These results suggest that patients with the AG genotype may develop osteoporosis.

## 1. Introduction

Hyperandrogenism (an excessive level of androgens in the female body) is a serious health problem affecting 5–10% of women of reproductive age. In total, 80% of hyperandrogenism is associated with PCOS (polycystic ovary syndrome), whereas in about 15%, it is impossible to associate hyperandrogenism with any disease entity (idiopathic hyperandrogenism). In adolescence, especially in the first years after the menarche, dysfunction of the hypothalamus may occur due to excessive psycho-emotional stress and physical exertion or due to the increasing scope of responsibilities (e.g., schoolwork). Behavioral disorders, eating disorders, or adolescent depression that occur at this time affect the neurosecretion of the hypothalamus to such an extent that they can lead to secondary amenorrhea [1]. The long-term effect of the psychogenic factor is one of the causes of functional hypothalamic amenorrhea (FHA). The frequency of this disorder is estimated at 2.6–8.5%; however, in the case of girls exposed to chronic stress, e.g., due to competitive training in certain sports, the incidence of FHA may be as high as 100% [2,3]. Other causes of excessive androgenization in women of reproductive age are ovarian tumors, adrenal tumors, and non-classical congenital adrenal hyperplasia (NCAH) [4]. The most common direct cause of hyperandrogenism is the excessive production of androgens in the ovaries or adrenal glands. Sometimes clinical signs of hyperandrogenism are observed with normal serum androgen concentrations [5]. In the female body, testosterone and androstenedione are converted into estrogens, estradiol, and estrone [6].

The enzyme responsible for converting androgens to estrogens is aromatase. This enzyme belongs to the cytochrome P450 family and is encoded by the *CYP19A1* gene located on chromosome 15q21.2 [7,8]. Reduced activity of this enzyme, due to mutations in the *CYP19A1* gene, reduces the estrogen pool in favor of androgens. Clinically, aromatase deficiency causes hyperandrogenism in women. Symptoms of aromatase deficiency are progressive virilization (change in body structure, hirsutism, alopecia). Girls do not develop secondary sexual characteristics properly during puberty [9]. The *CYP19A1* polymorphism is related to bone mineral density (BMD), and rs700518 is one of the most studied polymorphisms in association with BMD and osteoporosis [10,11].

It is now known that hyperandrogenism is a multi-gene disorder influenced by epigenetic and environmental factors. The search for genetic determinants aims to improve diagnostics and therapeutic possibilities. The aim of the study was to assess the effect of the *CYP19A1* gene polymorphism on selected markers of bone metabolism and hormonal parameters in women with hyperandrogenism, and whether the presence of polymorphisms of this gene may be a clinically useful marker in the prevention and treatment of disorders resulting from the hormonal disorders associated with hyperandrogenism. A further aim was to examine what role aromatase gene polymorphisms may have in the pathogenesis of hyperandrogenism in young women. Explaining the genetic determinants of this type of disorder may be important for devising methods of treating hormonal disorders to prevent subsequent menstrual, metabolic, and bone metabolism disorders.

## 2. Materials and Methods

### 2.1. Patients

The study group consisted of 80 young women aged 18 to 35 with hyperandrogenismwith secondary functional amenorrhea (FHA)attending the Department of Endocrinology, Metabolic Diseases, and Internal Diseases at the Pomeranian Medical University in Szczecin (Poland).The inclusion criteria for the genetic research were as follows: (1) Caucasian race, (2) at least six months of amenorrhea preceded by at least three years of oligomenorrhea, (3) no current history of chronic pharmacotherapy, and (4) a lack of major abnormalities upon physical examination. The exclusion criteria were: (1) polycystic ovary syndrome, congenital adrenal hyperplasia, or premature ovarian failure diagnosed on the basis of medical history, gynecological exam, and laboratory tests, (2) low birth weight or preterm birth, (3) at least one confirmed episode of an eating disorder, (4) poor diet during childhood or puberty, (5) episodes of impaired growth and body mass gain, (6) extensive participation in sports that may have influenced bone mineralization, (7) metabolic disorders that may be associated with decreased bone mineralization, (8) prolonged use of stimulants or drugs that may affect bone metabolism, and (9) a family history of osteoporosis.

The study was approved by the Ethical Committee of the Pomeranian Medical University (No. KB-0012/115/15). It was conducted in accordance with the Helsinki Declaration (1975, revised 2000). Written informed consent was obtained from all the participants.

### 2.2. Determination of Serum Concentrations for Selected Parameters

Blood samples were obtained in a fasted state at 8 a.m. and centrifuged immediately. Enzyme immunoassays (ELISA–DRG International, Inc., Springfield, NJ, USA) were used to measure total sRANKL (free and bound RANKL), osteoprotegerin, and a total of 25-OH Vitamin D.

Serum concentrations of parathyroid hormone and calcitonin were determined using the chemiluminescent method (Immulite 1000, Siemens, Tarrytown, NY, USA). Electrochemiluminescence immunoassays (Cobas, Roche Diagnostic, Santa Clara, CA, USA) were used to measure follicle stimulating hormone (FSH), luteinizing hormone (LH), sex hormone-binding globulin (SHBG), testosterone (T), androstenedione, 17-hydroxyprogesterone, estradiol, prolactin (PRL), dehydroepiandrosterone sulfate (DHEA-SO4), glucose, and insulin. The free androgen index (FAI = TT/SHBG × 100%) was used to evaluate free testosterone concentrations.

The analytical sensitivity of the tests was as follows: parathyroid hormone 3.1 pg/mL, calcitonin 2.0 pg/mL, 25-OH vitamin D total 5.6 nmol/L, osteoprotegerin 0.14 pmol/L, leptin 2.0 ng/mL, and sRANKLt 0.5 pmol/L. Elisa tests assays were performed as per the manufacturer’s instructions and were quality controlled using the manufacturer’s two-level control set. Samples were run in duplicate. The performance of the microplate reader and microplate washer used for the ELISA determinations and precision was controlledchecked with the Pathozyme Elisa Sure kit (Omega Diagnostics, Littleport, UK).

### 2.3. Bone Mineral Density (BMD) Measurements

Bone mineral density (BMD) measurements were taken at the Endocrinology Clinic at Clinical Hospital No.1, Pomeranian Medical University in Szczecin. BMD was measured in the lumbar spine from the L2 to L4 vertebrae with the use of DEXA (Dual Energy X-ray Absorptiometry), which means the use of double-beam radiation, i.e., simultaneous measurement with a high- and low-energy beam (GE LunarProdigyAdvance, Madison, WI,; enCORE software version 8.8). Densitometry was performed using a LUNAR DPX 100 camera (Lunar Corp., Madison, WI, USA).

In all the patients, the quantitative body weight composition (i.e., total body fat (BF), android, and gynoid fat and visceral adipose tissue (VAT), and lean body mass were measured using the DXA method using an automatic whole body scan method. The original manufacturer’s software (Body Composition) was used to determine the individual regions of measurements (female, male, and visceral region).

### 2.4. Analysis of the CYP19A1 rs700518 Polymorphism

Blood samples were collected at the Department of Endocrinology, Metabolic Diseases, and Internal Diseases, Pomeranian Medical University. *CYP19A1* genotyping was performed in the Clinical Laboratory at the Department of Endocrinology, Metabolic Diseases, and Internal Diseases, Pomeranian Medical University. Genomic DNA was extracted from peripheral blood using a QIAamp Blood Mini Kit (Qiagen GmbH, Hilden, Germany) according to the manufacturer’s protocol. The *CYP19A1* rs700518 polymorphism was selected as per the NCBI SNP database http://www.ncbi.nlm.nih.gov/SNP (accessed on 25 January 2021) and determined by Real-Time PCR using LightCycler 480 and TaqMan*^®^*SNP Genotyping Assays (Thermo Fisher Scientific, Waltham, MA, USA). Real-time PCR was performed in a 20 μL reaction mixture according to the manufacturer’s protocol, with initial denaturation at 95 °C for 10 min and 40 cycles as follows: denaturation at 95 °C for 15 s, annealing at 60 °C for 1 min, and cooling at 40 °C for 30 s.

All methods were carried out in accordance with the relevant guidelines and regulations.

### 2.5. Statistical Analysis

All the statistical analyses for this study were performed using SPSS Statistics 17.0 for Windows. The Chi-square test was used to calculate that the genotype prevalence and allele frequencies were in the Hardy–Weinberg equilibrium. We evaluated the effect of the *CYP19A1* polymorphism on selected biochemical and clinical parameters. Analysis for data distribution was performed using one-way analysis of variance (ANOVA). Values normally distributed were expressed as means ± SEM (standard error of mean).

## 3. Results

The frequency of genotypes for the *CYP19A1* rs700518 polymorphism in women with hyperandrogenism was compared. A higher frequency of the AG genotype (40.0%) was observed compared to the AA (36.2%) and GG (23.8%) genotypes (Table 1). Analysis of the rs700518 polymorphism showed a higher FEI (Free Estradiol Index) value in women with the AG genotype (FEI: GA–8.85 ± 1.18 vs. AA–7.38 ± 1.29, GG–3.30 ± 0.86, *p* = 0.015) (Table 2).

In the participants with the GG genotype, the estradiol concentration was the lowest (Estradiol (pg/mL): GG–34.53 ± 4.12 vs. AA–56.48 ± 8.11, AG–60.62 ± 8.12, *p* = 0.037). An association with SHGB concentration was also observed, with the highest values found in the GG genotype (SHGB (nmol/L): GG–61.50 ± 10.02 vs. AG–37.43 ± 8.60, AA–34.31 ± 3.73, *p* = 0.036) (Table 3). BAI (Body Adiposity Index) and androstenedione were lowest in the group with the GG genotype (BAI%: GG–33.77 ± 3.51 vs. AA–45.04 ± 2.63, AG–45.89 ± 2.64, *p* = 0.011; androstenedione (ng/mL): GG–3.76 ± 0.32 vs. AG–3.89 ± 0.19, AA–4.39 ± 0.51, *p* = 0.011) (Table 3).

However, the most significant of these parameters was the insulin 60’; in this case, the lowest concentration value was also observed for the GG genotype (insulin 60’ (μIU/mL): GG–84.67 ± 13.52 vs. AA–145.63 ± 11.01, AG–152.56 ± 18.27, *p* = 0.007) (Table 4).

For the indicators of adipose tissue distribution, i.e., AG (distribution of android and gynoid fat) and VF (Visceral Fat Indication), an association was found for the genotypes studied: the GG genotype was associated with lower values of AG and VF (AG: GG–0.90 ± 0.07 vs. AA–1.07 ± 0.05, AG–1.15 ± 0.05, *p* = 0.014; VF: GG–609.601 ± 206.95 vs. AA–719.06 ± 163.57, AG–1260.00 ± 197.81, *p* = 0.043) (Table 4). The differences in 25–OH Vitamin D concentration in the individual groups of genotypes were at the border of significance (Vitamin 25 (OH) D (ng/mL): GG–25.33 ± 1.53 vs. AA–21.39 ± 1.60, AG–20.00 ± 1.12, *p* = 0.05) (Table 5).

Concentrations of the remaining parameters of the *CYP19A1* rs700518 polymorphism: 17-OH-progesterone, LH, FSH, testosterone, FAI%, DHEA-SO4, glucose 0’, glucose 60’, glucose 120’, insulin 0’, insulin 120’, vitamin D levels, calcitonin, PTH, OPG, sRANKL, BMD total, BMD L1–L4, T-score, Z-score, BMI, BMC, and TBS in relation to the distribution of individual genotypes (AA, AG, GG) showed no statistically significant differences (Table 6).

## 4. Discussion

The CYP19 gene codes for the aromatase enzyme that converts androgens into estrogens. Any change in the expression of the *CYP19A1* gene may alter the levels of androgens and estrogens in the female body. It was found that normal aromatase activity is essential for the proper development of ovarian follicles during fetal development, their maturation, and during ovulation in the reproductive period. A deficiency of aromatase in most cases leads to the development of a polycystic ovary phenotype [12]. The *CYP19A1* rs700518 polymorphism is based on the conversion of adenine (A) to guanine (G) and is one of the most widely studied polymorphisms in relation to bone mineral density (BMD) and osteoporosis. Such a change may affect the post-transcriptional regulation of the gene, leading to a change in its expression and, consequently, changes in the concentration of aromatase and androgens [13,14].

Many authors have observed that *CYP19A1* gene mutations are associated with BMD and their presence may contribute to the development of osteoporosis [10,15]. Riancho et al. and Kaminski et al. showed that total hip BMD is higher in patients with the GG genotype compared to the GA and AA genotypes [15,16].

In a meta-analysis by Hua-Jing Ma, it was demonstrated that the AG genotype of the rs700518 polymorphism is associated with low BMD and plays a role in the development of osteoporosis. These studies are in line with our results, which indicate that the AG genotype is most associated with the risk of osteoporosis [17].In addition, Kaminski et al. demonstrated a higher frequency of the AG genotype compared to GG and AA in women with postmenopausal osteoporosis [15]. However, the results of the studies are often ambiguous, and few have demonstrated a significant effect of the rs700518 polymorphism on BMD [18,19].

Aguirre et al. observed that the expression of the *CYP19A1* gene was highest in the group with the rs700518 SNP polymorphism in the GG genotype [20]. In our study, the analysis of the rs700518 polymorphism of the *CYP19A1* gene showed an association of FEI on the GG genotype vs. AG and AA. The FEI value is the ratio of the estradiol concentration to the SHGB concentration. In our study, we also found a relationship between the concentration of SHGB and estradiol (components of the FEI formula) on the genotype of GG vs. AG and AA). With the GG genotype, the concentrations of SHGB were higher and the concentration of estradiol was lower. Ebrahimi et al. observed a strong correlation between the GG genotype of the rs700518 polymorphism and a lower estradiol concentration compared to the AA genotype in all the women studied. The authors showed a relationship between the severity of acne vulgaris and the frequency of the GG genotype of the polymorphism studied [21].

The level of sex hormones influences the distribution of adipose tissue. Estrogens are responsible for the fat distribution typical of women, which accumulates mainly in the subcutaneous tissue around the thighs and buttocks [22,23]. In our group of women with hyperandrogenism, we observed an association of the adipose tissue distribution indices (BAI, AG, and VF) with the GG, AA, and AG genotypes of the *CYP19A1* rs700518 polymorphism. In women receiving aromatase inhibitors, Napoli et al. found a relationship between the increase in body fat on the GG genome for the rs700518 SNP in the *CYP19A1* gene, but did not observe a similar phenomenon for the AG and AA genomes [24]. In our study, the GG genotype was associated with the lowest VF value (Visceral Fat Index), which is a measure of the amount of visceral fat. Visceral adipose tissue (VAT) is considered to be the main risk factor for the development of certain cardiovascular, metabolic, oncological, and autoimmune diseases. VAT is also a risk factor for early death [25,26]. An increased volume of visceral fat leads to a reduction in bone mineral density (BMD). In turn, a reduction in BMD represents an increased risk of developing osteoporosis. [17].Additionally, the Body Adiposity Index (BAI%) in the women we studied showed an association with individual genotypes: the lowest value was observed for the GG genotype, and the highest for AG. The distribution of the android and genoid fat (AG) index indicates the male (abdominal) or female type of fat distribution in the body. In our study of the rs700518 polymorphism, among women with hyperandrogenism, the AG index value was lowest in the GG genotype group and highest in the AG genotype group. Summarizing the influence of the *CYP19A1* rs700518 polymorphism in our group of hyperandrogenic women, we observed an association between the homozygous GG genotype and the amount and distribution of adipose tissue. Women with the GG genome had the lowest BAI% and the lowest percentage of adipose tissue in total body weight; moreover, its distribution was the most consistent with the female pattern adipose tissue distribution, and AG and VF rates were the lowest. The group of women with the GG genotype also had the lowest average body weight. Another interesting observation was the finding of a strong relationship between the GG genotype and the lowest insulin level 1 h after the 75 g oral glucose tolerance test (OGTT). The relationship between aromatase activity, glucose metabolism, and insulin sensitivity has been noted by many researchers. Chen et al. noticed a decrease in glucose concentration in OGTT in a patient with aromatase deficiency after the administration of estrogens. This confirms that aromatase activity lowers the level of glycemia by influencing the level of estrogens. These researchers, however, did not find that estradiol improved insulin resistance; after 6 months administering 25 μg of transdermal estradiol (Estraderm TTS, Novartis) twice a week, the fasting insulin level was 2.66 times higher than at the beginning of the study [27,28]. In our study, we observed an association between the GG genotype and lower insulin 60’ levels, but we did not observe significant differences in glucose levels in OGTT for individual genotypes.

In our study, the GG genotype was associated with the lowest content of visceral adipose tissue and the lowest ratio of android to gynoid adipose tissue. It has been proven that adipose tissue as an additional endocrine organ is a source of adipokines, hormones, cytokines, and other substances that adversely affect carbohydrate metabolism, including those increasing the risk of insulin resistance [29].

The influence of the *CYP19A1* rs700518 polymorphism on the bone metabolism of hyperandrogenic women was assessed by examining the following parameters: BMC—Bone Mineral Content, TBS—Trabecular Bone Score, BMD L1–L4—Bone Mineral Density L1–L4, T-score—the ratio of the bone mineral density (BMD) of the participant to the average bone density of a young person, Z-score—bone mineral density index, and BMD total—Bone Mineral Density total. Oz et al. confirmed the numerous occurrences of aromatase in bone tissue, chondrocytes, and osteoblasts [30]. On the other hand, Liu et al. demonstrated that the activity of aromatase encoded by the *CYP19A1* gene may have a significant impact on bone health, and women with lower activity of this enzyme may be more likely to have osteoporosis [31]. We did not observe statistically significant differences between the above parameters of bone metabolism between individual genotypes. Only a clear difference was observed for the Z-score, but it was not statistically significant. These Z-scores mean that the bone density in the GG group was closer to the average for a given age, and the women from the AA group had higher bone density than the average for a given age; an approximate Z-score = 0.1 corresponds to the 50th percentile and Z-score = 0.3 corresponds to the 60th percentile.

## 5. Conclusions

The study showed that the *CYP19A1* gene rs700518 polymorphism may be associated with the distribution of adipose tissue in young women with hyperandrogenism. The data showed that the GG genotype of the *CYP19A1* rs700518 polymorphism is associated with the lowest FEI, the highest SHGB, and the highest estradiol concentration and insulin concentration determined in the glucose tolerance test 60’ compared to the AG and AA genotypes.

On the other hand, the distribution of adipose tissue in women is an important factor affecting BMD, which may be associated with the risk of osteoporosis. Patients with the AG genotype had a significantly higher ratio of android to gynoid fat and a greater content of visceral adipose tissue. Higher visceral tissue content may reduce BMD. Patients with the AG genotype may develop osteoporosis. It seems that the CYP19A1 rs700518 polymorphism may be a potential marker used in the early prevention, diagnosis, and therapy of osteoporosis. However, more research is needed, involving more patients.

## Figures and Tables

**Table 1:** The frequency of alleles and genotypes of the *CYP19A1* polymorphism in women with hyperandrogenism.

*CYP19A1* rs700518		
Genotype	Observed Value n (%)	Expected Value (%)
AA	29 (36.20)	31.64
AG	32 (40.00)	49.22
GG	19 (23.80)	19.14
Total	80 (100)	100
Allele		
A	90 (56.25)	-
G	70 (43.75)	-
Total	160 (100)	-

The expected value was calculated in accordance with the Hardy–Weinberg equilibrium (HWE).

**Table 2:** Analysis of female hormonal parameters in relation to *CYP19A1* gene polymorphisms in patients with hyperandrogenism.

Parameter	CYP19A1 rs700518	Mean ± SEM	*p*
Estradiol (pg/mL)	AA	56.48 ± 8.11	0.037
	AG	60.62 ± 8.12	
	GG	34.53 ± 4.12	
FEI (pmol/nmol)	AA	7.38 ± 1.29	0.015
	AG	8.85 ± 1.18	
	GG	3.30 ± 0.86	
Prolactin (ng/mL)	AA	17.09 ± 1.95	0.613
	AG	16.28 ± 1.34	
	GG	20.57 ± 6.35	
17-OH-Progesterone (ng/mL)	AA	1.64 ± 0.37	0.341
	AG	1.31 ± 0.17	
	GG	1.04 ± 0.12	
LH (mIU/mL)	AA	8.99 ± 1.10	0.474
	AG	10.33 ± 1.52	
	GG	12.41 ± 3.28	
FSH (mIU/mL)	AA	7.71 ± 2.08	0.757
	AG	8.14 ± 2.47	
	GG	11.00 ± 5.36	

FEI—Free Estradiol Index, LH—Luteinizing Hormone, FSH—Follicle Stimulating Hormone. *p* < 0.05—comparison between genotypes and the parameters analyzed (one-way ANOVA test); values normally distributed are expressed as means ± SEM.

**Table 3:** Analysis of hormonal parameters in relation to *CYP19A1* gene polymorphisms in patients with hyperandrogenism.

Parameter	CYP19A1 rs700518	Mean ± SEM	*p*
Testosterone (ng/mL)	AA	0.51 ± 0.05	0.880
	AG	0.48 ± 0.03	
	GG	0.47 ± 0.07	
BAI%	AA	45.04 ± 2.63	0.011
	AG	45.89 ± 2.64	
	GG	33.77 ± 3.51	
FAI%	AA	1.92 ± 0.11	0.549
	AG	1.96 ± 0.11	
	GG	1.44 ± 0.15	
Androstendione (ng/mL)	AA	4.39 ± 0.51	0.011
	AG	3.89 ± 0.19	
	GG	3.76 ± 0.32	
DHEA-SO4 (μg/dL)	AA	248.29 ± 27.60	0.700
	AG	266.44 ± 30.59	
	GG	285.60 ± 34.35	
SHBG (nmol/L)	AA	34.31 ± 3.73	0.036
	AG	37.43 ± 8.60	
	GG	61.50 ± 10.02	

BAI%—Body Adiposity Index, FAI%—Free Androgen Index, DHEA-SO4—Dehydroepiandrosterone Sulfate, SHGB—Sex Hormone Binding Globulin. *p* < 0.05—comparison between genotypes and the parameters analyzed (one-way ANOVA test); values normally distributed are expressed as means ± SEM.

**Table 4:** Analysis of clinical parameters in relation to *CYP19A1* gene polymorphisms in patients with hyperandrogenism.

Parameter	CYP19A1 rs700518	Mean ± SEM	*p*
Glucose 0’ (mg/dL)	AA	88.88 ± 1.86	0.279
	AG	95.39 ± 4.74	
	GG	88.95 ± 1.74	
Glucose 60’ (mg/dL)	AA	137.40 ± 6.23	0.353
	AG	136.86 ± 6.35	
	GG	125.29 ± 6.94	
Glucose 120’ (mg/dL)	AA	107.79 ± 5.06	0.995
	AG	108.54 ± 4.32	
	GG	108.43 ± 7.41	
Insulin 0’ (μIU/mL)	AA	20.42 ± 2.85	0.074
	AG	35.00 ± 8.50	
	GG	16.52 ± 3.43	
Insulin 60’ (μIU/mL)	AA	145.63 ± 11.01	0.007
	AG	152.56 ± 18.27	
	GG	84.67 ± 13.52	
Insulin 120’(μIU/mL)	AA	99.04 ± 17.40	0.158
	AG	93.78 ± 10.33	
	GG	60.79 ± 15.01	
AG	AA	1.07 ± 0.05	0.014
	AG	1.15 ± 0.05	
	GG	0.90 ± 0.07	
VF	AA	719.06 ± 163.57	0.043
	AG	1260.00 ± 197.81	
	GG	609.601 ± 206.95	

AG—Distribution of android and gynoid fat, VF—Visceral Fat Index. *p* < 0.05—comparison between genotypes and the parameters analyzed (one-way ANOVA test); values normally distributed are expressed as means ± SEM.

**Table 5:** Analysis of bone metabolism parameters and clinical parameters in relation to *CYP19A1* gene polymorphisms in patients with hyperandrogenism.

Parameter	CYP19A1 rs700518	Mean ± SEM	*p*
Vitamin D (ng/mL)	AA	15.09 ± 3.16	0.056
	AG	11.86 ± 1.44	
	GG	21.40 ± 3.27	
25-OH Vitamin D (ng/mL)	AA	21.39 ± 1.60	0.050
	AG	20.00 ± 1.12	
	GG	25.33 ± 1.53	
Calcitonin (pg/mL)	AA	3.52 ± 1.98	0.374
	AG	1.27 ± 0.14	
	GG	1.86 ± 0.37	
PTH (pg/mL)	AA	39.15 ± 4.04	0.275
	AG	45.12 ± 4.41	
	GG	35.04 ± 4.09	
OPG (pmol/L)	AA	3.39 ± 0.21	0.471
	AG	3.78 ± 0.27	
	GG	3.73 ± 0.27	
sRANKL (pmol/L)	AA	262.56 ± 52.38	0.241
	AG	188.85 ± 18.19	
	GG	190.51 ± 18.91	

PTH—Parathyroid hormone, OPG—Osteoprotegerin, BMI—Body Mass Index. *p* < 0.05–comparison between genotypes and the parameters analyzed (one-way ANOVA test); values normally distributed are expressed as means ± SEM.

**Table 6:** Analysis of bone metabolism parameters in relation to *CYP19A1* gene polymorphisms in patients with hyperandrogenism.

Parameter	CYP19A1 rs700518	Mean ± SEM	*p*
BMD total	AA	1.18 ± 0.03	0.169
	AG	1.22 ± 0.02	
	GG	1.16 ± 0.02	
BMD L1–L4	AA	1.22 ± 0.03	0.366
	AG	1.25 ± 0.03	
	GG	1.19 ± 0.03	
T-score	AA	0.34 ± 0.32	0.267
	AG	0.24 ± 0.24	
	GG	0.27 ± 0.27	
Z-score	AA	0.32 ± 0.29	0.861
	AG	0.21 ± 0.28	
	GG	0.10 ± 0.18	
BMI	AA	27.85 ± 1.45	0.055
	AG	32.63 ± 2.03	
	GG	26.27 ± 2.19	
BMC (g)	AA	2403.84 ± 62.69	0.299
	AG	2452.41 ± 41.97	
	GG	2328.36 ± 57.87	
TBS	AA	1.34 ± 0.04	0.361
	AG	1.40 ± 0.03	
	GG	1.34 ± 0.03	

BMC—Bone Mineral Content, BMD L1–L4—Bone Mineral Density L1–L4, T-score—the ratio of the bone mineral density (BMD) of the participant to the average bone density of a young person, Z-score—bone mineral density index, BMD total—Bone Mineral Density total, TBS—Trabecular Bone Score. *p* < 0.05—comparison between genotypes and the parameters analyzed (one-way ANOVA test); values normally distributed are expressed as means ± SEM.

## Data Availability

Not applicable.
